# Becoming poor and the cutback in the demand for health services in Israel

**DOI:** 10.1186/2045-4015-2-49

**Published:** 2013-12-19

**Authors:** Joseph Deutsch, Adi Lazar, Jacques Silber

**Affiliations:** 1 Department of Economics, Bar-Ilan University, 52900 Ramat-Gan, Israel

## Abstract

**Background:**

This paper examines whether individuals facing the threat of poverty are curtailing their consumption of various goods and services in a given order and, if among the expenditures that are cut back, there are also health expenditures. The location of individuals in this order of cutback is then used to derive the degree of their deprivation and the factors that affect the extent of this deprivation.

**Methods:**

This order of curtailment of expenditures is obtained on the basis of an algorithm originally devised to derive the order of acquisition of durable goods. Having found the order of curtailment of expenditures on the basis of the 2003 Israel Social Survey, we then estimate an ordered logit regression whose latent dependent variable is assumed to measure the individual degree of deprivation.

**Results:**

The results of this estimation show that, other things constant, the individual latent level of deprivation increases with the size of the household, first increases and then decreases with the age of the individual, is higher when the individual has children under the age of five, has a low educational level, a low income, and when he/she is separated or divorced. Finally, deprivation is found to be lower among individuals with good health.

**Conclusion:**

Discovering the order of curtailment of expenditures, including health expenditures, of individuals facing economic difficulties and finding the determinants of the extent of such deprivation should help policy makers focus their attention on the population subgroups that are most likely to curtail their health expenditures when facing economic difficulties.

## Background

Evaluating the extent of poverty is not an easy task. In fact, during the past decades, one has observed a shift from a traditional approach emphasizing a single indicator of poverty (like income or consumption) to a broader view taking into account quite a long list of attributes assumed to characterize living conditions. The first to take such a position were (Townsend [1] and Sen [2]) who both argued that the level of income refers only to one aspect of well-being. They both recommended exploring a larger range of indicators of well-being (material and non-material) in order to better assess the living conditions of households.

At the same time, selecting a multidimensional approach to poverty implies addressing issues that need not be faced when taking a uni-dimensional approach. Thus, in addition to the problem of determining a poverty line and choosing a measure of poverty, a multidimensional approach forces the researcher to select the relevant dimensions of poverty, the indicators supposed to represent these dimensions and finally the weights to be given to these various dimensions and indicators. (Townsend [1]) thus made a distinction between relative deprivation and poverty and defined the latter as a situation of relative deprivation due to the lack of command over resources. The counting approach of Townsend was then refined in various works such as that of (Mack and Lansley [3]), (Halleröd [4] and Nolan and Whelan [5]) as well as (Atkinson [6]), (Alkire and Foster [7] and Aaberge and Peluso [8]).

More recently quite a few quantitative approaches to the study of multidimensional poverty have appeared in the literature (see, Silber, [9], for a survey of the multidimensional approaches to poverty measurement, and Kakwani and Silber, [10], for a detailed presentation of many of these approaches). Not many studies tried to compare the results obtained on the basis of the same data when using different multidimensional approaches. Using Israeli data, (Silber and Sorin [11]) compared three different methodologies all based on the so-called fuzzy set approach to multidimensional poverty measurement while (Deutsch and Silber [12]), using Israeli census data, made a systematic comparison of results based on four different approaches to multidimensional poverty: the fuzzy set approach previously mentioned, efficiency analysis, information theory and the axiomatic approach to multidimensional poverty measurement.

The present paper also takes a multidimensional approach to poverty measurement. It tries to better understand the decisions made by individuals facing the risk of becoming poor and emphasizes the types of expenditures which they decide to limit, especially those that are likely to affect their health. The idea is that there may well be some specific order in the type of expenditures which are curtailed. For example, do individuals give up some kind of medications before they decide to stop receiving medical treatment? Or, when getting poorer, do individuals give up health insurance before they curtail their demand for dental care?

To get an answer to these questions we adopt an approach taken recently by (Deutsch and Silber [13]) in their study of the link between multidimensional poverty and the order of acquisition of durable goods. The idea (for more details, see, Paroush, [14,15]) is that, in general, households acquire durable goods in a given order and that the stage in this order in which each household is located says something about its wealth. Alternatively, and this is the novelty of this paper, one can look at the decision made by households (individuals) to curtail the consumption of various goods and services and check whether there is some order in this impoverishment process. However, such a curtailment is likely to reflect both the degree to which a family regards some good as desirable and the degree to which the good is liquid (i.e., the degree to which it can be converted into cash through sale or avoidance of use^a^). Both effects are likely to have an influence on a household’s (individual’s) degree of deprivation which will be estimated via a latent variable to be derived from this order of curtailment. One may then derive the impact of various explanatory variables on the degree of (relative) deprivation of the different households/individuals.

The next section describes the original ideas of (Paroush [14-16]) concerning an order of acquisition of durable goods. Then we show how this same idea may be applied to the process of impoverishment and how, using an ordered Logit or Probit regression, it is possible to estimate a latent variable which reflects the degree of deprivation of each individual or household. The paper ends with an empirical illustration based on data from the 2003 Israeli Social Survey.

### The concept of an order of acquisitions of durable goods^b^

Assume we collect information on the ownership of three durable goods A, B and C. A household can own one two, three or none of these goods. There are therefore 2^3^ = 8 possible profiles of ownership of durable goods in this example. Table 1 summarizes the various possibilities. A number 1 indicates that the household owns the corresponding good, a zero that it does not. If we assume that the order of acquisition is A, B, C (that is, that a household first acquires good A, then good B and finally good C) there would be no household with the profiles 3, 4, 6 and 7. More precisely, for a given order of acquisition and k durable goods there are k + 1 possible profiles in the acquisition path. We do not want to assume however that every household has to follow this order A, B, C. There are always households that deviate slightly from this most common order of acquisition.

**Table 1:** A list of possible orders of acquisition when there are 3 goods

**Ownership profile**	**The household owns good A**	**The household owns good B**	**The household owns good C**
1	0	0	0
2	1	0	0
3	0	1	0
4	0	0	1
5	1	1	0
6	0	1	1
7	1	0	1
8	1	1	1

Paroush suggested computing the minimum number of changes in numbers (from 0 to 1 or from 1 to 0) necessary to bring a deviating household back to one of the profiles corresponding to a given order of acquisition of durable goods. We can compare the profile of a given individual *i* with every possible profile in the acquisition path and define the deviation of the profile of individual *i* as the distance of the profile of individual *i* to the closest profile in the acquisition path (for more details, see, Deutsch and Silber, [13]). The complement to one of the weighted average of these individual deviations, which is a kind of proximity index, has been called “coefficient of reproducibility” (see, Paroush, [14-16]). However, we do not know what the most common order of acquisition in the population is. We have to discover it. We therefore have to compute the proximity index previously defined for each possible order of acquisition. If there are *k* goods we know that there are *k*! such profiles. The most commonly selected order of acquisition in the population will then be the one with the highest value of the proximity index.

### The derivation of a deprivation index

The concept of order of acquisition of durable goods may also be applied to the reverse situation, where one wishes to find out in which order individuals or households curtail their expenditures when they face a serious decrease in their income or wealth. The 2003 Israeli Social Survey that we used in our empirical illustration provides quite detailed information on the consumption of various goods and services related to health, but we will also consider other elements which may crucial in the process of impoverishment.

However, one should remember that curtailment is a time-varying process: the process of curtailment is one in which families make decisions on a continual basis about what to cut next in their budgets. Using a cross-sectional survey like the 2003 Israeli Social Survey and examining the set of goods that a family reports to have given up in the prior twelve months provides only a very rough and unreliable snapshot of which goods they gave up first, second, and third. Trying to estimate the curtailment parameter by looking at the general ordering of having/not having those goods tells us more about the clustering between different forms of deprivation than about the order under which those deprivations took place. This problem is amplified because deprivation is treated as a latent variable. Without directly observing the exposure to a health or non-health shock, it is very difficult to know how much that shock led to a particular order of curtailment. Families face a multitude of different hardships, and the nature of hardships may determine the order of curtailment. For example, a family with very young children and inadequate income may curtail certain medical expenses before food, but an elderly family facing a sudden health shock may give greater priority to prescription medications over food. One should hence be very careful in linking the complex and multifaceted nature of poverty to a measure of curtailment^c^.

Keeping in mind these caveats we now turn to the questions in the Social Survey which we considered as relevant for the purpose of our study:

•Did you forgo dental work in the past 12 months because you could not afford it?

•Did you forgo buying prescription drugs in the past 12 months because you could not afford it?

•Did you forgo receiving any medical treatment (other than dental work or prescription drugs) in the past 12 months because you could not afford it?

•Is the reason you do not have additional health insurance coverage because you cannot afford it?

•In the past 12 months, did you forgo adequately heating or cooling your dwelling because you could not afford it?

•In the past 12 months, did you forgo buying clothing or shoes because you could not afford it?

•In the past 12 months, was your electricity or phone service disconnected because you could not afford to pay your bills?

•In the past 12 months, did you sometimes not eat because you did not have enough money?

As stressed previously, the main idea here is that, instead of talking about an order of acquisition of durable goods, one can try to discover the sequence of restrictions that individuals follow in an impoverishment process which leads them to limit their use of health services. The analysis of this curtailment process should help us understand how families balance their budgets inter-temporally, but one should keep in mind the fact that a study of this type cannot by itself reveal the relative value that households place on goods. We nevertheless believe that discovering some general patterns in foregone consumption among households that are believed to be under financial strain is likely to shed important light on the impoverishment process^d^.

The analysis is the same as in the case of the acquisition of durable goods except that we now focus on the first good or service a household prefers to give up, the second one and so on. Accordingly, in the above example of three durable goods A, B and C, the interpretation now is that a household/individual can give up one, two, three or none of these items. There are as before 2^3^ = 8 possible profiles of restrictions and the number 1 (zero) in the previous table will now indicate that the household gives up (does not give up) the corresponding item. The order of curtailment of expenditures will therefore be defined as the one with the highest value of the proximity index defined previously.

Following the determination of a most common order of curtailment of the demand for health services, an ordered logit^e^ procedure is used to better understand the factors affecting this order, or more generally the factors having an impact on the level of deprivation. The idea, following (Paroush [14-16]), is to assume that the stage in which a household is located in the order of expenditures restrictions says something about its level of deprivation.

Let *D*_*i*_ denotes the level of deprivation of household *i* such that a higher value of *D*_*i*_ corresponds to higher degrees of deprivation. Such a deprivation score is assumed to be a function of *H* factors whose value for household *i* is *X*_*ih*_, *h* = 1 to *H*. We may therefore express this latent variable *D*_*i*_ as

$$D_{i}=\sum_{h=1}^{H}\beta_{h}X_{\mathrm{ih}}+\epsilon_{i}$$

Such a deprivation level is however not observed. What is assumed is that this deprivation level is related to the stage in the “curtailment process” in which the household/individual is located. The parameters of such a regression are estimated using the ordered logit procedure. It is then possible to compute the probability that a given household with characteristics *X*_*ih*_ belongs to one of the profiles defined by the “order of curtailment” (for more details, see, for example, Deutsch and Silber, [13]).

### Empirical results based on the 2003 Israel social survey

#### The Israel Central Bureau of Statistics (ICBS) social survey

This survey has been conducted annually since 2002 on a sample of persons aged 20 and older. Its main purpose is to provide up-to-date information on the welfare of Israelis and on their living conditions. The social survey questionnaire has two main parts: a core questionnaire containing about 100 items covering the main areas of life such as details of household members, fertility, dwelling, ownership of cars, household help, religion, employment, economic situation, skills (studies, languages, courses, use of computers and the internet), relations with family and friends, volunteering, victimization, subjective attitudes on well-being and standard of living regarding health, housing, region, economic situation and satisfaction in general. Each year there is also a varying module devoted to one or two special topics. In 2003 the special topic was the multidimensional measurement of welfare.

The questionnaires are administered by ICBS interviewers using laptops to conduct computer-assisted personal interviews. The interviews are conducted in Hebrew, Arabic and Russian, with about 7500 people aged 20 and over, who represent about 4.5 million people in that age bracket. The interview lasts about an hour. The survey population comprises the permanent non-institutional population of Israel aged 20 and older, as well as residents of non-custodial institutions (such as student dormitories, immigrant absorption centers and independent living projects for the elderly). New immigrants are included in the survey population if they have been present in Israel for at least six months. The groups not included in the survey population are the residents of custodial institutions (e.g. old-age homes, hospitals for the chronically ill, prisons), Israelis abroad for more than a year without interruption at the time of the survey, diplomats, new immigrants who arrived fewer than six months prior to the interview, Bedouins and other persons living outside the boundaries of localities.

#### The empirical investigation

Using the information available on the eight types of expenditures that were selected, we applied several times the algorithm described previously. Each time we analyzed a somehow different set of expenditures because if we had analyzed simultaneously the eight types of expenditures we would have been left with too few observations, given the relatively high degree of non-response on some of the questions. The first time we included six types of expenditures (the ones mentioned previously at the exception of “another medical treatment” and “additional health insurance”). It then turns out that the order of curtailment of expenditures with the highest proximity index is the one given in the second column of Table 2.

**Table 2:** Order of curtailment with the highest proximity coefficient (First illustration)

**Number of curtailed expenditures**	**Which additional expenditure is curtailed**	**Corresponding percentage of individuals**
0	None	44.6%
1	Clothing or Shoes	19.9%
2	Dental Work	8.9%
3	Heating or Cooling	13.4%
4	Food	4.5%
5	Prescription Drugs	4.4%
6	Electricity or Phone	4.3%

The proportion of individuals having this profile of curtailment of expenditures is around 64% (1837 individuals)^f^. For this set of variables, the second stage of the empirical analysis will therefore be based only on these 1837 individuals.

In the second illustration, seven types of expenditures were included (all the types except “additional health insurance”). We then found that the order of curtailment of expenditures with the highest proximity index was that given in the second column of Table 3.

**Table 3:** Order of curtailment with highest proximity coefficient (Second illustration)

**Number of curtailed expenditures**	**Which additional expenditure is curtailed**	**Corresponding percentage of individuals**
0	None	40.1%
1	Clothing or Shoes	18.3%
2	Heating or Cooling	9.7%
3	Dental Work	15.0%
4	Food	4.5%
5	Another Medical Treatment	1.8%
6	Prescription Drugs	4.4%
7	Electricity or Phone	6.2%

In this illustration, the proportion of individuals with a profile of curtailment of expenditures corresponding to the different stages of the order of curtailment given in Table 3 is around 58% (682 individuals)^g^. The second stage of the empirical analysis referring to this set of expenditures will therefore be based on only these 682 individuals.

In the third and last illustration, we included also seven types of expenditures (all of them except “another medical treatment”). We then found that the order of curtailment of expenditures with the highest proximity index is the one given in the second column of Table 4.

**Table 4:** Order of curtailment with highest proximity coefficient (Third illustration)

**Number of curtailed expenditures**	**Which additional expenditure is curtailed**	**Corresponding percentage of individuals**
0	None	32.6%
1	Clothing or Shoes	10.7%
2	Dental Work	4.9%
3	Additional Health Insurance	5.5%
4	Heating or Cooling	15.2%
5	Prescription Drugs	8.5%
6	Food	10.1%
7	Electricity or Phone	12.5%

In this case, the proportion of individuals with a profile of curtailment of expenditures corresponding to the different stages of the order of curtailment given in Table 4 is around 49% (328 individuals)^h^. The second stage of the empirical analysis referring to this set of expenditures will therefore be based on only these 328 individuals.

It is interesting to note that if we derive the order of curtailment without including expenditures on health services, the order obtained which has the highest proximity index is the one given in Table 5. The proportion of individuals having this profile of curtailment of expenditures is around 84%.

**Table 5:** Order of curtailment with the highest proximity coefficient of the non-health services expenditures

**Rank**	**Goods and services**
1	Clothing or Shoes
2	Heating or Cooling
3	Food
4	Electricity or Phone

If we now add the expenditures on health services and implement one of the three illustrations described in Tables 2 to 4, the order of curtailment of the non-health services expenditures remains unchanged. In other words, the order of cutback of the demand for non-health services is not affected by the various health services that are included.

In the ordered logit regression^i^ that was described previously, the following exogenous variables were available in the 2003 Social Survey and have been taken into account: the age of the individual and its square^j^, the gender, the household size, the highest diploma received (one dummy variable for elementary school and another dummy for post secondary education), the area of residence of the individual (one dummy variable for Jerusalem), the marital status (one dummy variable for separated or divorced), the country of birth of the individual and his/her father’s country of birth (one dummy variable for those born in Israel with a father born also in Israel, one dummy for those born in Israel with a father born in Europe or America, one dummy for those born in Asia or Africa and another dummy for those born in Europe or America), religion (one dummy variable for the Jews), the health status of the individual (one dummy variable for good health), the income level of the household (one dummy for high income and another dummy for low income) and the presence of young children (a dummy variable equal to 1 when there are children less than six years old).

These results are given in Tables 6, 7 and 8. It appears that the explanatory variables that were introduced have generally a significant impact. Thus individuals who have a higher educational level have, ceteris paribus, a lower level of deprivation. Household income has a similar impact on the level of deprivation. Other things constant we also observe that the level of deprivation is lower among Jews, among those who live in Jerusalem and among males. Ceteris paribus this level of deprivation is also lower when the individual enjoys good health.

**Table 6:** Results of the ordered logit regression for the first illustration (Dependent Variable = Latent variable measuring the level of deprivation)

**Explanatory Variable**^**m**^	**Coefficient**	**Std. Error**	**Wald**^**n**^	**Sig.**
Age	0.117	0.020	34.345	0.000
Square of Age	-0.001	0.000	49.092	0.000
Household Size	0.162	0.033	23.654	0.000
Male	-0.702	0.094	55.863	0.000
Separated/Divorced	0.763	0.172	19.764	0.000
Jews	-0.429	0.173	6.111	0.013
Elementary School	0.683	0.248	7.555	0.006
Post Secondary Education	-0.422	0.098	18.351	0.000
Jerusalem	-0.302	0.160	3.531	0.060
Born in Israel and the father was born in Israel	-0.467	0.135	11.910	0.001
Born in Israel but the father was born in Europe or America	-0.562	0.134	17.702	0.000
Asia/Africa	0.369	0.161	5.232	0.022
Children aged 0-5	0.336	0.120	7.827	0.005
High Income	-1.200	0.122	96.713	0.000
Low Income	1.557	0.158	97.427	0.000
Good Health	-1.315	0.129	103.382	0.000

Notes: Number of observations: 1837.

Pseudo R-square: 0.1216.

Log-Likelihood:-2546.62^0^.

**Table 7:** Results of the ordered logit regression for the second illustration (Dependent Variable = Latent variable measuring the level of deprivation)

**Explanatory Variable**^**p**^	**Coefficient**	**Std. Error**	**Wald**	**Sig.**
Age	0.091	0.031	8.766	0.003
Square of Age	-0.001	0.000	15.555	0.000
Household Size	0.251	0.057	19.668	0.000
Male	-0.529	0.150	12.382	0.000
Separated/Divorced	0.861	0.247	12.138	0.000
Jews	-0.428	0.238	3.225	0.073
Elementary School	0.816	0.360	5.146	0.023
Post Secondary Education	-0.535	0.168	10.127	0.001
Born in Israel but the father was born in Europe or America	-0.508	0.281	3.261	0.071
Asia/Africa	0.913	0.283	10.414	0.001
Europe/America	0.502	0.203	6.120	0.013
Children aged 0-5	0.393	0.215	3.337	0.068
High Income	-1.062	0.208	26.092	0.000
Low Income	1.523	0.221	47.353	0.000
Good Health	-1.054	0.177	35.591	0.000

Notes: Number of observations: 682.

Pseudo R-square: 0.1245.

Log-Likelihood: -1019.97.

**Table 8:** Results of the ordered logit regression for the third illustration (Dependent Variable = Latent variable measuring the level of deprivation)

**Explanatory Variable**^**q**^	**Coefficient**	**Std. Error**	**Wald**	**Sig.**
Age	0.092	0.039	5.475	0.019
Square of Age	-0.001	0.000	7.731	0.005
Household Size	0.134	0.072	3.418	0.065
Male	-0.567	0.212	7.170	0.007
Jews	-0.586	0.285	4.230	0.040
Elementary School	0.920	0.512	3.228	0.072
Jerusalem	-0.909	0.356	6.498	0.011
Born in Israel but the father was born in Europe or America	-1.199	0.508	5.573	0.018
Asia/Africa	1.092	0.415	6.904	0.009
Children aged 0-5	0.520	0.288	3.254	0.071
High Income	-1.521	0.454	11.224	0.001
Low Income	1.353	0.271	24.983	0.000
Good Health	-1.715	0.288	35.350	0.000

Notes: Number of observations: 328.

Pseudo R-square: 0.1251.

Log-Likelihood: -544.84.

The results of the ordered logit regression also show that the level of deprivation first increases and then decreases with the age of the individual, the estimated turning points being 39 years in the first illustration, 36 in the second and 39 again in the third illustration^k^. Such a non-monotonic effect of the age may be explained by the fact that at intermediary ages parents are likely to have the highest number of children depending financially on them. One should remember here that the only information available concerns the presence of small children and the total size of the household but not the number of children.

The level of deprivation is also higher when the individual was born in Europe or America and even higher when he/she was born in Asia or Africa. The level of deprivation increases also with the size of the household, when the household has children less than six years old and when the individual is separated or divorced^l^.

As mentioned previously these empirical results are based on a survey conducted a decade ago and a priori one might wonder whether they are still relevant today. It is however important to remember first that data sources combining information on the wealth of individuals, or at least on important aspects of their standard of living, as well as on decisions they had to make concerning their health when facing economic hardship are not commonly available. The SHARE (Survey of Health, Ageing and Retirement in Europe) may include, at least partly, this type of information but it covers only individuals who are at least 50 years old. Second the empirical findings of the present study seem to be corroborated by more recent studies of health inequity in Israel.

(Epstein et al. [17]), for example, who analysed the results of a survey conducted by the Smokler Center of Health Policy Research, observed that the respondents to this survey had “identified a set of related population characteristics as major influences on overall health status and the development of health inequality. Those cited as being most influential were education, income, area of residence, ethnic origin, religion and degree of religious observance…”. The respondents indicated also “that unemployment and poor health can constitute a self-perpetuating cycle. When unemployment is at very high levels, people are reluctant to spend time going to a physician or taking sick leave. With incomes reduced, the unemployed are also more affected by co-payments for physician visits and medications. Reluctance to visit a physician can also be compounded by a lack of reasonably-priced transportation, which can cause an individual to forgo ambulatory or hospital treatment for himself or his child. Parents (especially mothers) face the additional financial burden of obtaining child care when they themselves require medical care”.

Quite similar conclusions were drawn on the basis of surveys conducted by the Israeli Medical Association (see, Degani and Degani [18,19]). According to the survey conducted in 2009, the proportion of individuals who are forced to give up prescription drugs is higher among individuals with a low socio-economic level than among those coming from higher socio-economic strata. This survey shows that in 2008 the proportion of individuals forced to give up prescription drugs because of their high price was 17% among those with a low socioeconomic background, 12% among those belonging to the middle class and 10% among individuals having a high socioeconomic level. Similar results were obtained on the basis of the survey conducted in April 2008 by the Israeli Medical Association since it showed that the proportion of those giving up prescription drugs was higher in peripheral areas than in the center of the country (which is on average richer than the periphery).

Another survey conducted by the Brookdale Institute (see, Brammli-Greenberg et al., [20]) confirmed that the proportion of individuals forgoing prescription drugs in 2007 was particularly high among low-income groups (19%). Half of those forgoing prescription drugs even indicated that they did so for a chronic illness. This survey also found that 12% of the respondents had gone without medical treatment or medication or both. Among individuals with a low-income, the proportion rose to 20%. These findings confirm thus the results presented previously which showed that giving up medical treatment is a sign of high degree of poverty.

## Conclusion

The goal of this paper was to assess (using an algorithm previously applied to detect the order of acquisition of durable goods) whether individuals facing the threat of poverty were curtailing their consumption of various goods and services in a given order and, whether among the expenditures that were cut back or even suppressed, there were health expenditures. Using the 2003 Israel Social Survey we found that dental work was among the first expenditures to be curtailed. Using an ordered logit regression we then estimated for each individual a latent variable measuring his/her degree of deprivation. We observed that, other things constant, the level of deprivation increased with the size of the household but first increased and then decreased with the age of the individual. We also found that this level of deprivation was higher when the individual had children under the age of five, had a low educational level, a low income and when he/she was separated or divorced. Deprivation was also found to be lower among individuals with a good health. It thus appears that the cutback in health expenditures is an important signal of the impoverishment process. Such a conclusion should have important policy implications if one accepts the principle according to which the right to a decent health should be one of the basic human rights of individuals.

## Endnotes

^a^ We thank a referee for drawing our attention to this important point.

^b^ This section is based on (Deutsch and Silber [21]).

^c^ Here again we are extremely thankful to a referee who drew our attention to these issues. The two last paragraphs are largely based on his/her remarks.

^d^ Once more we acknowledge here the very insightful remarks of a referee on which the last paragraph is largely based.

^e^ We could have also used an ordered probit model.

^f^ Note that in this illustration there were 306 missing values among the explanatory variables.

^g^ Here there were 153 missing values among the explanatory variables.

^h^ There were 115 missing values among the explanatory variables.

^i^ The regression was estimated for each illustration mentioned previously.

^j^ The age of the individual is actually the average age in each range of ages.

^k^ For the result of the non-monotonic effect of the age, see, Deutsch and Silber, [12].

^l^ We should stress here that by controlling for social and demographic factors in the regression analysis, these factors should be considered as capturing the likelihood of experiencing a higher order of deprivation, and not a particular order of deprivation. It certainly would be also very interesting to examine which of the demographic factors predicts a particular sequence of curtailment. We leave such an analysis for future work. We thank a referee for making such a suggestion.

^m^ For schooling there are two dummy variables-one for elementary school and another for post secondary education. The excluded categories refer to intermediate education. For the place of birth there are three dummy variables-one for those born in Israel with a father born also in Israel, one for those born in Israel with a father born in Europe or America and another for those born in Asia or Africa. For income there are two dummy variables-one for high incomes, consisting of the two highest income categories (namely, incomes over 15,000 NIS) and another for low incomes, consisting of the two lowest categories (namely, incomes up to 3000 NIS). The excluded categories refer to incomes over 3000 NIS and up to 15,000 NIS.

^n^ This is the Wald chi-square test that tests the null hypothesis that the coefficient (parameter) equals 0. This hypothesis is rejected when the p-value (listed in the column called “Sig.”) is smaller than the critical p-value of 0.05 (or 0.01).

^o^ The log likelihood reflects how likely it is (the odds) that the observed values of the dependent may be predicted from the observed values of the independent variables.

^p^ For schooling there are two dummy variables-one for elementary school and another for post secondary education. The excluded categories refer to intermediate education. For the place of birth there are three dummy variables-one for those born in Israel with a father born in Europe or America, one for those born in Asia or Africa and another for those born in Europe or America. For income there are two dummy variables-one for high incomes, consisting of the two highest income categories (namely, incomes over 15,000 NIS) and another for low incomes, consisting of the two lowest categories (namely, incomes up to 3000 NIS). The excluded categories refer to incomes over 3000 NIS and up to 15,000 NIS.

^q^ For schooling there is one dummy variable which is equal to 1 for elementary school. The excluded categories refer to intermediate and post secondary education. For the place of birth there are two dummy variables-one for those born in Israel with a father born in Europe or America and another for those born in Asia or Africa. For income there are two dummy variables-one for high incomes, consisting of the two highest income categories (namely, incomes over 15,000 NIS) and another for low incomes, consisting of the two lowest categories (namely, incomes up to 3000 NIS). The excluded categories refer to incomes over 3000 NIS and up to 15,000 NIS.

## Competing interests

The authors declare they have no competing interests.

## Authors’ contributions

The authors jointly prepared the final version of this paper. JD was mainly in charge of devising the algorithm allowing discovering the most common order of curtailment of expenditures. AL focused her attention on the econometric analysis and wrote the first draft of the paper. JS was in charge of preparing the final version of the paper, focusing his attention in particular on the introduction and the conclusion. All authors read and approved the final manuscript.

## Authors’ information

Joseph Deutsch is currently the chair of the Department of Economics at Bar-Ilan University. He holds a Ph.D. from Bar-Ilan University and has published numerous papers in the field of poverty and inequality measurement.

Adi Lazar obtained her Ph.D. at Bar-Ilan University in 2012 and is a CPA. She teaches at Bar-Ilan University and published recently with Jacques Silber an article in *Health Economics*.

Jacques Silber is Professor Emeritus at Bar-Ilan University. He was (2011-2013) the President of the Society for the Study of Economic Inequality ECINEQ) and was the Founder and First Editor-in-Chief of the *Journal of Economic Inequality*.

## References

[B1] Townsend P Poverty in the United Kingdom: a survey of household resources and standards of living 1979 Harmondsworth: Penguin

[B2] Sen A Commodities and Capabilities 1985 Amsterdam: North Holland

[B3] Mack J Lansley S Poor Britain 1985 London: George Allen and Uniwin

[B4] Halleröd B A new approach to the direct consensual measurement of poverty 1994 Sydney Australia: University of New South Wales, Social Policy Research Centre, discussion paper no. 50

[B5] Nolan B Whelan CT Resources deprivation and poverty 1996 Oxford: Oxford University Press

[B6] Atkinson AB Multidimensional deprivation: contrasting social welfare and counting approaches J Econ Inequal 2003 2 1 51 65 10.1023/A:1023903525276

[B7] Alkire S Foster J Counting and multidimensional poverty measurement J Public Econ 2011 2 7-8 476 487 10.1016/j.jpubeco.2010.11.006

[B8] Aaberge R Peluso E A counting approach for measuring multidimensional deprivation Discussion paper No. 700 2012 Research Department, Statistics Norway

[B9] Silber J Measuring poverty: taking a multidimensional perspective Hacienda Pública Española 2007 2 3 29 73

[B10] Kakwani N Silber J Quantitative approaches to multidimensional poverty measurement 2008 New York: Palgrave Macmillan

[B11] Silber J Sorin M Petmesidou M, Papatheodorou C Poverty and social deprivation in the Mediterranean Area: trends, policies and welfare prospects in the new millennium Poverty in Israel: taking a multidimensional approach 2006 London: Zed Books / CROP Series

[B12] Deutsch J Silber J Measuring multidimensional poverty: an empirical comparison of various approaches Review of Income and Wealth 2005 2 1 145 174 10.1111/j.1475-4991.2005.00148.x

[B13] Deutsch J Silber J Kakwani N, Silber J Quantitative approaches to multidimensional poverty measurement The order of acquisition of durable goods and the multidimensional measurement of poverty 2008 New York: Palgrave-Macmillan 226 243

[B14] Paroush J The order of acquisition of durable goods Bank of Israel Survey 1963 2 47 61

[B15] Paroush J The order of acquisition of consumer durables Econometrica 1965 2 1 225 235 10.2307/1911897

[B16] Paroush J Efficient purchasing behavior and order relations in consumption Kyklos XXVI 1973 2 91 112

[B17] Epstein L Goldwag R Isma’il S Greenstein M Rosen B Reducing health inequality and health inequity in Israel: towards a national policy and action program The Smokler Center for Health Policy Research 2006 Jerusalem: The Myers-JDC-Brookdale Institute

[B18] Degani A Degani R “Consumption of health services in Israel-central versus peripheral,” survey of the Israeli medical association 2008

[B19] Degani A Degani R “The impact of the economic burden on the consumption of health services in Israel,” survey of the Israeli medical association 2009

[B20] Brammli-Greenberg S Gross R Waitzberg R Public opinion on the level of service performance of the health-care system in 2007 and in comparison with previous years 2009 The Myers-JDC-Brookdale Institute, Jerusalem: The Smokler Center for Health Policy Research

[B21] Deutsch J Silber J Ethnic origin and multidimensional relative poverty in Israel: a study based on the 1995 Israeli census Res Labor Econ 2006 2 235 264 10.1086/499972

